# A Novel Small *NPC1* Promoter Enhances AAV-Mediated Gene Therapy in Mouse Models of Niemann–Pick Type C1 Disease

**DOI:** 10.3390/cells12121619

**Published:** 2023-06-13

**Authors:** Michael Paul Hughes, Hemanth Ramesh Nelvagal, Oliver Coombe-Tennant, Dave Smith, Claire Smith, Giulia Massaro, Laura Poupon-Bejuit, Frances Mary Platt, Ahad Abdul Rahim

**Affiliations:** 1 UCL School of Pharmacy, University College London, London WC1N 1AX, UKh.nelvagal@ucl.ac.uk (H.R.N.); oliver.coombe-tennant.21@ucl.ac.uk (O.C.-T.); giulia.massaro.13@ucl.ac.uk (G.M.);; 2 Department of Pharmacology, University of Oxford, Oxford OX1 3QT, UK; dave.smith@pharm.ox.ac.uk (D.S.); claire.smith@pharm.ox.ac.uk (C.S.); frances.platt@pharm.ox.ac.uk (F.M.P.)

**Keywords:** Niemann–Pick type C disease, AAV, promoters, gene therapy, mouse model

## Abstract

Niemann–Pick disease type C1 (NP-C) is a prematurely lethal genetic lysosomal storage disorder with neurological and visceral pathology resulting from mutations in the *NPC1* gene encoding the lysosomal transmembrane protein *NPC1*. There is currently no cure for NP-C, and the only disease modifying treatment, miglustat, slows disease progression but does not significantly attenuate neurological symptoms. AAV-mediated gene therapy is an attractive option for NP-C, but due to the large size of the human *NPC1* gene, there may be packaging and truncation issues during vector manufacturing. One option is to reduce the size of DNA regulatory elements that are essential for gene expression, such as the promoter sequence. Here, we describe a novel small truncated endogenous *NPC1* promoter that leads to high gene expression both in vitro and in vivo and compare its efficacy to other commonly used promoters. Following neonatal intracerebroventricular (ICV) injection into the CNS, this novel promoter provided optimal therapeutic efficacy compared to all other promoters including increased survival, improved behavioural phenotypes, and attenuated neuropathology in mouse models of NP-C. Taken together, we propose that this novel promoter can be extremely efficient in designing an optimised AAV9 vector for gene therapy for NP-C.

## 1. Introduction

Niemann–Pick diseases are a subgroup of lysosomal storage disorders (LSDs) first described by Albert Niemann and Ludwig Pick in the early 20th century that can be further categorised by their respective lysosomal protein deficiency. Niemann–Pick Type A (NP-A) and B (NP-B) are caused by the loss of acid sphingomyelinase (ASM) function, whereas Type C1 and C2 result from the loss of either *NPC1* or *NPC2* function, respectively [1,2]. Niemann–Pick disease type C1 (NP-C) (OMIM 257220) results from mutations in the *NPC1* gene and is a prematurely lethal genetic lysosomal storage disorder (LSD). Although visceral organs including the liver and spleen are affected, the clinical course is dominated by progressive neurodegeneration in the brain leading to premature death [1,2]. The most prominent and earliest pathology is seen in the brain, resulting in neuron loss, especially that of the Purkinje cells in the cerebellum. The clinical onset of symptoms varies, but can be broadly classified into early infantile, late infantile, juvenile, and adult cases [1]. Progressive neurological symptoms include ataxia, cognitive decline, dementia, vertical gaze palsy, epilepsy, and dysphagia with subsequent aspiration pneumonia as a leading cause of death [3].

The *NPC1* gene encodes a 13-domain transmembrane protein that is localised to the membrane of the late endosomes and lysosomes and plays a central role in transporting cholesterol from these vesicular organelles into the membranes [4]. Mutations in *NPC1* lead to an accumulation of glycosphingolipids, sphingosine, sphingomyelin, and cholesterol in cells of the body. However, which of these metabolites leads to pathology is yet to be determined [5,6].

Various mouse models of NP-C are available for the characterisation of pathology and pre-clinical assessment of therapeutic modalities. The most extensively studied is the NPC1 null model (*Npc1^nih^* or *Npc1^m1n^*) that carries a spontaneous mutation and accumulates the hallmark cholesterol and sphingolipids [7] (https://www.informatics.jax.org/allele/summary?markerId=MGI:1097712, accessed on 11 June 2023). Neurodegeneration is observed in the cerebellum but also the thalamus, cortex, and substantia nigra and is accompanied by microglial and astrocyte activation [8]. The neurological symptoms that develop at 6 weeks of age (tremor and decline in locomotor function) reflect those seen in NP-C patients and the mice reach their humane endpoint at 9–10 weeks of age. An alternative mouse model of NP-C is the *Npc1^nmf164^* which carries a point mutation in a region of the *NPC1* gene where a high proportion of mutations are found in NP-C patients [9]. The biochemical, locomotor, and pathological phenotype is similar to that observed in *Npc1^nih^* but has a slower progression.

While there are ongoing efforts such as enzyme replacement therapy [10] and autophagic modulation [11] for NP-A and NP-B, the availability of reliable mouse models has led to the successful pre-clinical testing of different therapeutic modalities and their subsequent clinical trials. These include the heat shock protein arimoclomol (ClinicalTrials.gov Identifier: NCT04316637), hydroxypropyl-beta-cyclodextrin (NCT04860960, NCT03887533), N-Acetyl-L-Leucine (NCT03759639), HDAC inhibitor vorinostat (NCT02124083), and substrate reduction therapy using miglustat (NCT00517153). To date, miglustat is currently the only licensed drug for treating NP-C in some territories and is generally considered to have a disease modifying effect [12,13].

Following the recent regulatory approval and licensing of the adeno-associated viral vector (AAV)-based gene therapy Zolgensma for the treatment of spinal muscular atrophy [14,15], there is hope for this approach in other neurological diseases including NP-C. A number of pre-clinical studies, including those conducted by our group, have shown that the AAV9 vector is able to deliver a therapeutic copy of the *NPC1* gene to the brain of the *Npc1^nih^* mouse model leading to extension of lifespan, improved locomotor function, an amelioration of neuronal loss and neuroinflammation, and a reduction in cholesterol and glycosphingolipid accumulation [16,17,18,19]. This is encouraging given that there is no therapeutic advantage from the cross-correction of untransduced neighbouring cells, as NPC1 is membrane-bound [20]. Furthermore, the *NPC1* cDNA sequence is relatively large (3.8Kb final coding sequence, RefSeq: CCDS11878.1) in the context of the limited packaging capacity of AAV vectors (4.7 Kb) and restricts the selection of genetic regulatory sequences such as promoters and polyA signals, leading to possible packaging and truncation errors [21]. Therefore, despite the promising results of these preclinical studies, further optimisation of the vectors is required to rise to the challenge of having a therapeutic effect in the larger and more complex human brain.

The promoter sequence is a crucial DNA regulatory element in the viral vector genome that determines the level of transgene expression as well as tissue specificity. Often, a strong, constitutively expressed promoter is desired for the high-level expression of NPC1, as there is no possible cross-correction of this membrane-bound protein [20]. Commonly used promoters of this type include the CMV (cytomegalovirus) promoter/enhancer, EFS (elongation factor 1a), GAPDH (glyceraldehyde-3-phosphate dehydrogenase), mPGK (phosphoglycerate kinase), CBA (chicken β-actin), and CAG (chicken β-actin promoter with CMV enhancer). All these promoters provide constitutively active gene expression in most cell types [22,23]. We have previously described the efficacy of the neuron-selective human Synapsin1 (SYN) promoter in treating mouse models of neurodegenerative LSDs, including neuronopathic Gaucher disease [24] and NP-C [16]. However, as many of these promoters have sizes larger than 400 bp that may lead to packaging and truncation issues during AAV vector production, it is crucial to find a promoter that drives strong expression but is small in length for developing an optimal gene therapy strategy for NP-C.

In this study, we compared a broad range of promoters to identify one that is small enough while maintaining high-level gene expression of the large *NPC1* gene and remain within the optimal 4.7 Kb packaging capacity of AAV9. Ideally, this small promoter would also outperform other sequences commonly used in gene therapy studies and clinical trials to provide maximum levels of NPC1 expression and subsequent enhanced therapeutic efficacy. We found that a small, truncated version of the endogenous human *NPC1* promoter fulfilled these criteria and significantly enhanced the therapeutic efficacy in both the *Npc1^nih^* and *Npc1^nmf164^* mouse models. This optimised AAV vector provides a major step forward towards the effective clinical translation of gene therapy for NP-C.

## 2. Materials and Methods

### 2.1. Plasmid and Vector Production

Human *NPC1* cDNA (RefSeq CCDS11878.1) was cloned into an AAV construct containing the various promoter sequences (Supplementary File S2) and SV40 late polyadenylation signal sequence, flanked by AAV2 inverted terminal repeats.

Human Embryonic Kidney 293T (HEK293T) cells were triple transfected with the ITR-containing plasmid carrying the respective therapeutic transgene cassette, a helper plasmid expressing AAV2 Rep and AAV9 Capsid, and a third construct containing the adenovirus helper functions (HGTI) using 3.5 mg/mL DNA polyethylenimine MAX (Polysciences, Inc. Warrington, PA, USA) and a 1:1:3 ratio of the respective plasmids [25]. Cells were harvested by centrifugation 72 h post-transfection and lysed by three freeze–thaw cycles (−80 °C to 37 °C) with vortexing in a lysis buffer (150 mM NaCl, 50 mM Tris, pH 8.5). Subsequent benzonase treatment (Sigma, Dorset, UK) was followed by lysate, and was cleared by centrifugation at 3200 g for 30 min. Subsequently, iodixanol gradient purification was performed in ultracentrifuge tubes (Beckman Instruments, High Wycombe, UK) with the lysate overlaid on increasing layers of 15, 25, 40, and 60% iodixanol (OptiPrep; Sigma). The tubes were centrifuged (Sorvall Discovery 90SE) for 3 h at 200,000 g in a TH641 (ThermoScientific, Paisley, UK) rotor. The vector was extracted from the 40% fraction with a 19-gauge needle, diluted in sterile phosphate-buffered saline, filtered at 0.22 mm, and concentrated in a Vivaspin 20 with a 100,000 molecular weight cut-off (Sartorious Stedim Biotech, Epsom, UK) centrifugal concentrator.

The concentrated vector genome was determined using quantitative PCR, using primers to the SV40 polyA sequence. The integrity of the vector genome was assessed using alkaline agarose gel electrophoresis [26]. Briefly, alkaline gels were run with 0.05 M NaOH as the running buffer, stained post-electrophoresis with 4× GelRed stain (Biotium, Fremont, CA, USA), and quantified against a HyperLadder 1 kb (Bioline Reagents, London, UK). Further analysis of concentrated AAV particle titre and vector purity was also assessed by visualization of the capsid proteins VP1, VP2, and VP3 via a SYPRO Ruby (ThermoScientific) protein stain, after sodium dodecyl sulfate–polyacrylamide gel electrophoresis [27].

### 2.2. Cell Culture and Plasmid Transfection

HEK 293T cells were grown in a standard growth medium of Dulbecco’s modified Eagle’s medium (DMEM; Gibco, ThermoFisher Scientific, Waltham, MA, USA) with high glucose (4.5 mg/mL), GlutaMAX (0.8 mg/mL), and pyruvate (0.1 mg/mL) and supplemented with 10% heat-inactivated foetal bovine serum (FBS; Sigma-Aldrich), penicillin (100 units/mL), and streptomycin (100 µg/mL). All cells were grown on poly-D-lysine coated six-well plates, T75 and T175 flasks, and 100 mm dishes to maintain adherence (Nunc, ThermoFisher Scientific). Cells were incubated at 37 °C in 5% CO_2_ for an average of 48–72 h between passaging.

For transfecting plasmids, cells were seeded in either a six-well plate (Nunc) at a ratio of 200,000 cells/well or a twelve-well plate at 10,000 cells/well with Poly-D-Lysine (PDL) coated coverslips (Thermofisher) along with standard growth media and incubated for 24 h. Plasmid DNA was mixed with the transfection agent linear polyethylenimine MAX (PEI MAX Mw ~ 40,000, Polysciences Inc., Warrington, PA, USA) at a ratio of 1:3 in reduced serum media OptiMEM (Gibco). Transfection mix was incubated at room temperature for 15 min to allow DNA-PEI complex formation and was subsequently added to the cells in the standard growth media. After 24 h, the media was replaced with fresh standard growth media and incubated for a further 24–48 h, at which point cells were either imaged or collected for downstream protein analysis.

### 2.3. Primary Cortical Cell Culture of E15 Mouse Embryos

Pregnant CD1 mice were sacrificed by schedule 1 methods at 15 days of gestation and embryonic cortices prepared as described previously [28]. Briefly, cortices from embryos in a single litter were dissected, meninges were removed, and the tissue was pooled. Cortices were roughly chopped before incubation in 0.25% trypsin/EDTA followed by trituration. Cells were pelleted by centrifugation, resuspended, and plated in a Neurobasal medium supplemented with B27, 100 units/mL of penicillin, 100 μg/mL of streptomycin, 0.25 μg/mL of amphotericin B, 300 μM of glutamine, and 25 μM of 2-mercaptoethanol at a density of 2.106 cells/6 cm plate. Transfections with plasmids were carried out on cells grown for a minimum of 7 days in vitro.

### 2.4. Immunocytochemistry and Cellular Imaging

For imaging cells, cells grown and transfected on PDL-coated coverslips were fixed using 4% Paraformaldehyde added to culture media for 15 min at room temperature. Coverslips were washed five times with Phosphate Buffered Saline (PBS) and blocked with 10% Normal serum solution in 0.1% PBS with Triton-X (PBS-T). Coverslips were then incubated overnight in an anti-GFP antibody (Abcam, Cambridge, UK), ab290, 1:200) in 10% normal serum solution in PBS-T followed by three washes in PBS and 2 h incubation in a secondary antibody (goat anti-rabbit Alexa Fluor 488, 1:200, Life Technologies, Paisley, UK) and three washes in PBS. Coverslips were then counterstained with DAPI for nuclear visualization, mounted, and coverslipped with Fluoromount G (SouthernBiotech, Birmingham, AL, USA). Sections were visualized with a laser scanning confocal microscope (Zeiss LSM 710, Carl Zeiss AG, Cambridge, UK).

### 2.5. Animals

All animal studies were approved by the UK Home Office for the conduct of regulated procedures under license (Animal Scientific Procedures Act, 1986) and according to ARRIVE guidelines and recommendations. Wild-type CD-1 mice, as well as wild-type and *Npc1^nih/nih^* mice on *Npc1*^nih^ (BALB/c) and *Npc1^nmf164^* (C57Bl6/J) were maintained as individual colonies in approved biological service units at University College London under a 12 h light/dark cycle and provided food and water ad libitum. For survival, any mice that lost 15% of their body weight after a 24 h period were considered to have reached their humane endpoint and sacrificed.

### 2.6. Intracerebroventricular (ICV) Injections

Neonatal injections were carried out as previously described [27]. Briefly, viral vector preparations were injected into 1-day post-gestation (P1) neonatal mice via bilateral ICV injection targeting the anterior horn of the lateral ventricle using a 33-gauge needle (Hamilton, Reno, NV, USA). Injected neonates were subsequently returned to the dam. After appropriate duration post-injection, the mice underwent terminal exsanguination by trans-cardiac perfusion with phosphate-buffered saline. Brains and organs were subsequently extracted and either fixed in 4% paraformaldehyde for immunohistochemistry or snap frozen on dry ice and stored at −80 °C for protein analysis.

### 2.7. IVIS Imaging

Imaging for luciferase expression in vivo was performed as previously described [29]. Briefly, animals were anesthetized with isoflurane (Abbott Laboratories), injected intraperitoneally (I.P.) with firefly D-luciferin (15 mg/mL in PBS; Gold Biotechnology, St. Louis, MO, USA), and imaged 5 min later with a cooled charge-coupled device (CCD) camera (IVIS; PerkinElmer, Waltham, MA, USA). The luciferin dose was 150 mg/kg; the volume varied with the age/size of the animal. Grey-scale photographs were acquired with a 24 cm field of view and a bioluminescence image was obtained using a binning/resolution factor of 4, a 1.2/f stop, and open filter. Regions of interest (ROIs) were defined manually using a standard area for each organ under investigation. Signal intensities were calculated with Living Image software v4.0(Perkin Elmer) and expressed as photons/second/cm^2^/steradian.

### 2.8. Western Blotting

For cell culture experiments, 48–72 h post-transfection cells were collected for protein extraction. A total of 100 µL of cold RIPA lysis buffer (ThermoFisher Scientific) with a 1× protease inhibitor cocktail (ThermoFisher Scientific) was added per well and incubated on ice for 5 min, swirling the plate every minute. Cells were then scraped and collected at the bottom of the well and the lysate was transferred to 1.5 mL microcentrifuge tubes. Resulting lysates were incubated on ice for 30 min, after which debris was pelleted by centrifugation at 14,000× *g*, 4 °C for 20 min. Lysate was carefully extracted and the overall protein concentration was determined by Pierce BCA Protein Assay (LifeTechnologies (Carlsbad, CA, USA), ThermoFisher Scientific). Sample protein concentrations were standardised to 1 µg/µL to standardise the loading volumes.

For brain tissues, tissues were homogenized (Ultra-Turrax TP, IKA Labortechnik, Wasserburg, Germany) on ice in 300 mL of RIPA lysis buffer (Thermo) per 100 mg of tissue with a 1× protease inhibitor cocktail (Thermo) and incubated for 30 min. Lysates were centrifuged at 14,000× *g*, 4 °C for 20 min and the overall protein concentrations of the supernatant was determined by Pierce BCA Protein Assay (Life Technologies).

Samples were incubated at 37 °C for 30 min in a 1× LDS sample buffer (Life Technologies) and a 1× sample reducing agent (Life Technologies), after which 40 mg of protein were loaded per well in a NuPAGE Bis–Tris 4–12% polyacrylamide gel for protein separation via SDS-PAGE electrophoresis. Proteins were transferred to a PDVF membrane at 400 mA for 1 h and the membrane was blocked for 1 h at 4 °C with 5% BSA in Tris Buffered Saline (TBS) with 3% Tween-20. Membranes were subsequently incubated overnight at 4 °C with primary antibodies for NPC1 (1:10,000, ab134113, Abcam), b-tubulin (1:2000, ab6161, Abcam), or b-Actin (1:100, ab8227, Abcam) with 3% BSA in TBS with 3% Tween-20. After three washes in TBS, antibody staining was revealed using HRP-conjugated goat anti-rabbit IgG (1:2000, ab6721, Abcam) and goat anti-rat IgG (1:10,000, ab97057, Abcam) incubated for 2 h at room temperature in TBS 3% Tween-20 with 3% BSA. Blots were developed with the ECL system (SuperSignal West Pico, Life Technologies) and imaged using a Genegnome imager (Syngene, Cambridge, UK).

### 2.9. Behavioural Analysis

#### 2.9.1. Tremor

*Npc1^nih/nih^* mice demonstrate an age-dependent increase in 32–55 Hz high frequency tremors [5]. Tremor analysis was carried out on control, untreated, and treated *Npc1^nih/nih^* at 10 weeks of age, using a commercial tremor monitor (San Diego Instruments, San Diego, CA, USA) as previously described [30]. Individual mice were placed inside the apparatus on an anti-vibration table and monitored for 256 s, after 30 s of acclimatisation time. The output (amplitude/time) was analysed using LabView software, to give a measurement of power at each monitored frequency (0–64 Hz).

#### 2.9.2. Gait

Gait monitoring was performed on control, untreated, and treated *Npc1^nih/nih^* at 10 weeks of age by using the automated gait analysis CatWalk system (Noldus, Wageningen, The Netherlands). The mice were monitored by being individually placed at one end of the CatWalk and were filmed freely walking across a filmed section with a backlit stage. A minimum of five successful runs were recorded per session, where a run across the stage was deemed successful if standard run criteria were met. Paw prints during successful runs were checked, classified, and analysed using the CatWalk XT software v10.6 (Noldus) to produce overall run measurements. Parameters recorded included the duration of the run (seconds), stride length (the distance between successive paw placement of the same paw in cm), swing speed (speed of the paw between successive paw placement, cm/sec), and regularity index (% index for the degree of interlimb coordination during gait) [16].

### 2.10. Histological Tissue Processing and Analysis

After 48 h fixation in 4% PFA, the brains were transferred into 30% sucrose in phosphate-buffered saline for cryoprotection. The brains were then cryosectioned at 20 °C using a Leica CM3050 cryostat microtome to 20 µm thickness.

Briefly, a one in twelve series of coronal brain sections and one in six sagittal cerebellum sections from each mouse were stained on slides using a modified immunofluorescence protocol [31] for the following antibodies–astrocytes (mouse anti-GFAP, 1:200, Millipore MAB3402), microglia (rat anti-mouse CD68, 1:400, Bio-Rad MCA1957), LAMP1 (rabbit anti-LAMP1, 1:200, Abcam ab24170), and calbindin (Rabbit anti-calbindin, 1:2000, Swant CB38a). A total of 20 µm coronal sections were mounted on superfrost plus slides (Fisher Scientific) and air-dried for 30 min, and the slides were then blocked in a 15% serum solution (Normal goat serum, S-1000 Vector Laboratories) in 4% TBS-T (1× Tris Buffered Saline, pH 7.6 with 4% Triton-X100, Fisher Scientific) for 1 h. The slides were then incubated in a primary antibody in 10% serum solution in 4% TBS-T for 2 h. Slides were washed three times in 1× TBS and incubated in fluorescent Alexa-Fluor-labelled IgG secondary antibodies (Alexa-Fluor goat anti-rabbit 488 Invitrogen A-11008, goat anti-rat 546 Invitrogen A-11081, Alexa-Fluor goat anti-mouse 546 Invitrogen A-11003) in 10% serum solution in 4% TBS-T for 2 h, washed three times in 1× TBS, and incubated in a 1× solution of TrueBlack lipofuscin autofluoresence quencher (Biotium, Fremont, CA, USA) in 70% ethanol for 2 min before rinsing in 1× TBS. Slides were coverslipped in a fluoromount-G mounting medium with DAPI (Southern Biotech, Birmingham, AL, USA).

A 3,3′-diaminobenzidine (DAB, Sigma-Aldrich) mediated immunohistochemistry (IHC) was used for neuron counts with NeuN (mouse anti-NeuN, 1:500, Millipore MAB377) and cortical analyses. Free-floating brain sections were incubated in a 1% H_2_O_2_ solution for 30 min to block endogenous peroxidase activity followed by three washes with TBS. A block of non-specific binding was then performed by a 30 min incubation in 15% normal serum with 0.3% TBS-T. The solution was then changed to 10% normal serum in 0.3% TBS-T with the primary antibody and left to incubate overnight at 4 °C. The sections were washed three times with TBS followed by incubation in a biotinylated secondary antibody (anti-mouse, 1:1000, Vector Laboratories) in 10% normal serum with 0.3% TBS-T for 2 h. After another three washes with TBS, visualisation of the staining was achieved with a Vectastain avidin-biotin solution (Vector Laboratories) and DAB (Sigma). The sections were then mounted, dehydrated, cleaned in histoclear, and coverslipped with DPX (VWR).

To analyse the degree of immunofluoresence in cellular images as well as the brain sections, a semiautomated thresholding image analysis was used with Image-Pro Premier software v9.1 (Media Cybernetics, Rockville, MD, USA) [31]. For in vitro cultures, 30 non-overlapping images were collected for *n* = 3 samples, as described above, at a 40× magnification. For brain sections, slide-scanned images at 10× magnification for stained sections were collected for all animals using a Zeiss Axioscan Z1 (Zeiss Microscopy Deutschland GmbH, Oberkochen, Germany) followed by demarcation of the anatomical regions of interest. Images were subsequently analyzed using Image-Pro Premier (Media Cybernetics) using appropriate thresholds that selected the foreground immunoreactivity above the background. Separate thresholds were used for each antigen and each anatomical region analysed.

Cortical thickness was measured in brain sections stained with Anti-NeuN antibody using Stereo Investigator software (MBF Bioscience, Williston, VT, USA). This was done by drawing 10 individual contours over the entire thickness of the cortex across three separate sections and collecting the average value in µm.

Estimates of neuron populations in the cerebellum for Calbindin-positive Purkinje neurons were performed by manual stereological counts and normalised per 1000 µm. Counts in the barrel field of the primary somatosensory cortex (S1BF) and Ventral posteromedial/lateral nuclei (VPM/VPL) of the thalamus were performed using a design-based optical fractionator method in a 1 in 12 series of NeuN-stained sections using Stereo Investigator software (MBF Bioscience) [31]. Cells were sampled with counting frames (90 × 100 μm) distributed over a sampling grid (VPM/VPL, 350 × 350 μm; S1BF, 450 × 450 μm) that was superimposed over the region of interest at 100× magnification. 

### 2.11. Statistical Analysis

All statistical analyses were carried out with GraphPad Prism software (Version 9.0e). Multiple comparisons were analysed by one-way analysis of variance (ANOVA) followed by post hoc Bonferroni’s correction, or a two-way ANOVA post hoc Bonferroni’s correction. All graphs are plotted as the mean ± the standard deviation (SD), unless stated otherwise, and statistical significance was compared to controls and assumed for *p* < 0.05; ns—non-significant, * *p* < 0.05, ** *p* < 0.01, *** *p* < 0.001, and **** *p* < 0.0001. Full details of all tests are reported in Supplementary File S1.

## 3. Results

### 3.1. A Novel Truncated Endogenous NPC1 Promoter Is Effective in Driving eGFP Reporter Gene Expression

**Figure 1:**
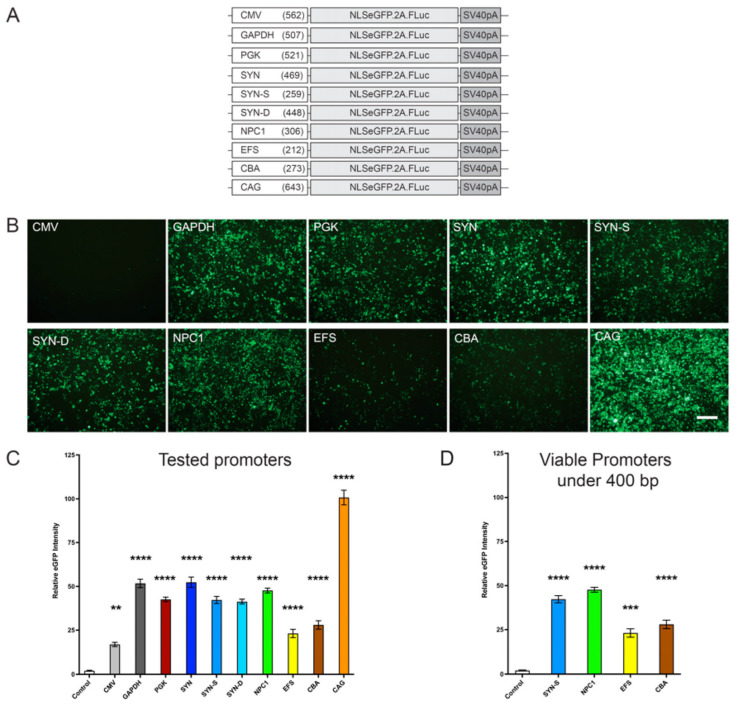
In vitro promoter comparison with NLSeGFP reporter gene. (**A**) Representative figures of different promoters tested with size in base pairs (bp). (**B**) Representative confocal fluorescence images taken 72 h post-transfection of plasmids containing selected promoters expressing nuclear localised eGFP in HEK293T cells. Scale bar = 200 µm. (**C**) Quantification of the relative eGFP intensity from transduced cells (*n* = 3 wells) for all promoters and (**D**) for promoters under 400 bp. One-way ANOVA with post-hoc Bonferroni correction, *n* = 3, error bars indicate ± SD. All significance shown is in comparison to untreated *Npc1^nih/nih^* control group. ** *p* < 0.01, *** *p* < 0.001, and **** *p* < 0.0001. A full list of *p*-values is reported in Supplementary File S1.

A range of promoter sequences were cloned into a bicistronic AAV expression plasmid that contained the enhanced green fluorescent protein (eGFP) fused to the SV40 nuclear localising sequence and the firefly luciferase gene (*FLuc*) (AAV-NLSeGFP.2A.FLuc) [16]. These promoters included CMV (562 bp), GAPDH (507 bp), PGK (521 bp), SYN (469 bp), SYN-S (Shortened Synapsin I promoter) (259 bp) [32,33], SYN-D (Neuron de-targeted Synapsin1) (448 bp) [32], NPC1 (307 bp), EFS (EF1a Core Promoter) (212 bp), CBA (273 bp), and CAG (643 bp) (Figure 1A) (See Supplementary File S2 for promoter sequences and map). To test functionality, the plasmids were transfected into HEK293 cells with untransfected cells as controls (*n* = 3). After 72 h, the eGFP expression was imaged (Figure 1B) and quantified (Figure 1C,D). All promoters provided significantly more expression of eGFP in the HEK293 cells compared to control untransfected cells, with CAG being the most efficient in this cell line (all *p*-values are shown in Supplementary File S1) (Figure 1C). GAPDH, SYN, and *NPC1* promoter sequences also provided robust levels of gene expression. Of the promoters under 400 bp, SYN-S and NPC1 showed the highest expression of eGFP (Figure 1D).

The strong neuronal promoter SYN and ubiquitous promoter CAG have both been used in gene therapy preclinical studies targeting the brain [14,16,24,34]. However, the *NPC1* promoter, in any form, has never been tested in the context of gene therapy studies. Therefore, to assess its expression profile in neural cells of the brain, viral preparations were produced of AAV9-SYN-NLSeGFP.2A.FLuc, AAV9-CAG-NLSeGFP.2A.FLuc, and AAV9-NPC-NLSeGFP.2A.FLuc. Primary cortical cell cultures were prepared from the brains of E18 wild-type mice embryos and transduced with the three viral vectors at a multiplicity of infection (MOI) of 100,000 vg/cell. To confirm the cell types in which expression is taking place, the transduced cell cultures were labelled by immunofluorescence using antibodies against eGFP, the neuronal specific antibody NeuN, and the glial astrocyte specific antibody GFAP. Cell nuclei were labelled using a DAPI stain. Scanning confocal microscopy revealed that eGFP expression was seen in cells using all three vectors (Supplementary Figure S1). All three vectors provided strong eGFP expression in cells that were positive for the neuronal nuclear marker NeuN. AAV9-CAG-NLSeGFP.2A.FLuc and AAV9-NPC-NLSeGFP.2A.FLuc both also provided eGFP expression in astrocytes labelled with antibodies against GFAP. AAV9-SYN-NLSeGFP.2A.FLuc did not produce any expression in astrocytes.

### 3.2. Efficient NPC1 Promoter Mediated Reporter Gene Expression in the Brains of Wild-Type Mice following Intracerebroventricular Administration of Vector

**Figure 2:**
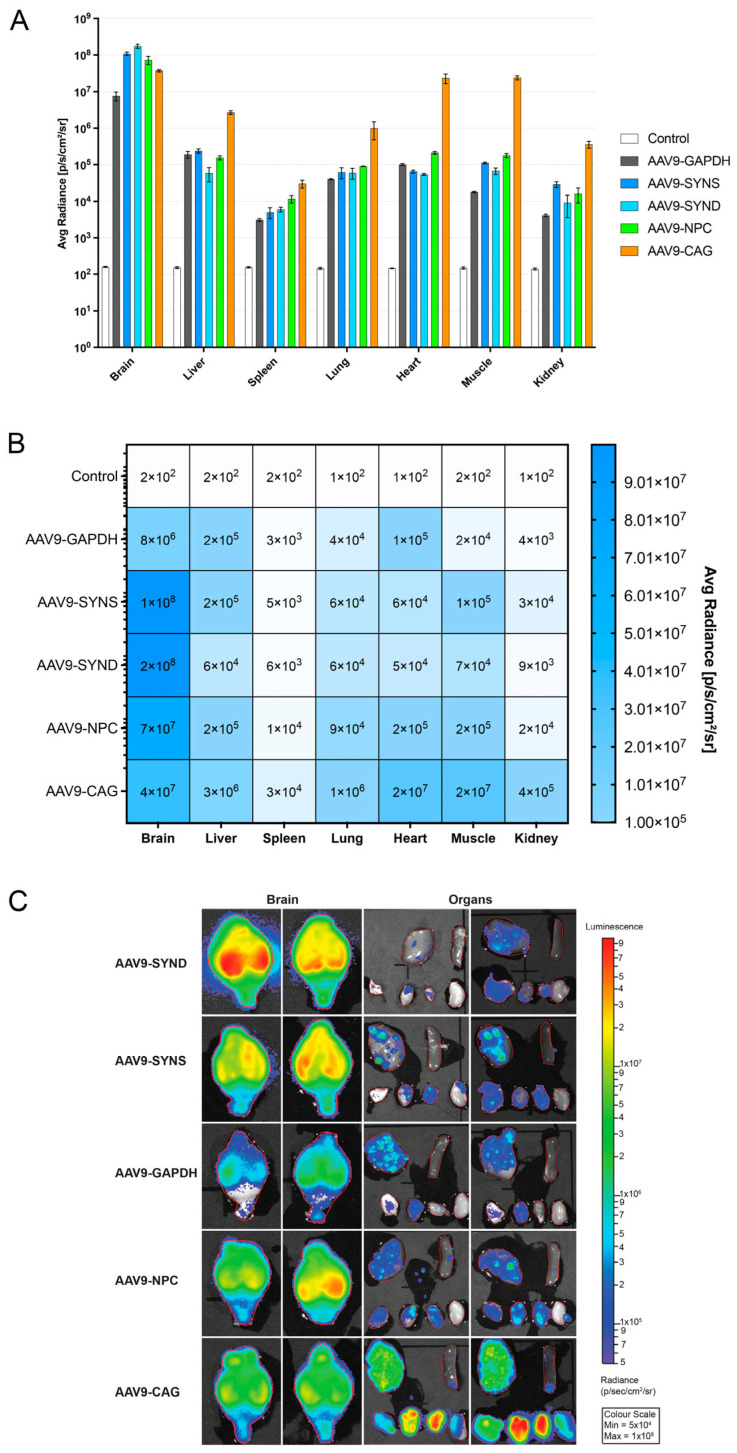
In vivo promoter comparison with luciferase reporter gene. Comparison of luciferase expression in brains and peripheral organs of P50 wild-type CD-1 mice administered ICV neonatally with AAV9.NLSeGFP.2AFLuc under control of different promoters. (**A**) Quantification of luciferase expression levels from promoters in analysed tissue in photons/second/cm^2^/steradian. Two-way ANOVA with post-hoc Bonferroni correction, *n* = 3, error bars indicate ± SD. A full list of *p*-values is reported in Supplementary File S1. (**B**) Heatmap of quantified luciferase expression levels in analysed tissue and (**C**) representative images of brains and visceral organs from each group.

To compare the ability of the different promoters to express AAV-NLSeGFP.2A.FLuc in vivo, we generated the following viral preparations—AAV9-CAG-NLSeGFP.2A.FLuc, AAV9-GAPDH-NLSeGFP.2A.FLuc, AAV9-SYN-NLSeGFP.2A.FLuc, AAV9-SYN-D-NLSeGFP.2A.FLuc, and AAV9-NPC1-NLS*eGFP*.2A.*FLuc*. All vectors were titer-matched and 1 × 10^11^ vg (10 µL at 1 × 10^13^ vg/mL) were administered into the brains of neonatal wild-type P0–1 CD-1 mice via intracerebroventricular injection. Age-matched, un-injected mice acted as controls (*n* = 3 per experimental cohort). Fifty days post injection, luciferin was administered intraperitoneally to the mice before being culled, and organs including the brain, liver, spleen, lung, heart, skeletal muscle, and kidney were harvested and then imaged using the in vivo imaging system (IVIS) [29]. All vectors produced significantly more luciferase expression measured as average radiance (p/sec/cm^2^/sr) in all organs compared to un-injected controls (Figure 2A–C) (all *p*-values are shown in Supplementary File S1). The highest levels of expression in the brain were achieved using AAV9 with the SYN-D, SYN-S, and *NPC1* promoters. Lower levels of expression in the brain were achieved using the CAG promoter, and lower still using the GAPDH promoter. The significant expression in the peripheral organs suggested escape of the vector from the CSF in systemic circulation. However, the CAG promoter provided the highest levels of expression in all peripheral organs.

### 3.3. Analysis of a Range of Promoters in Driving Human NPC1 Gene Expression

**Figure 3:**
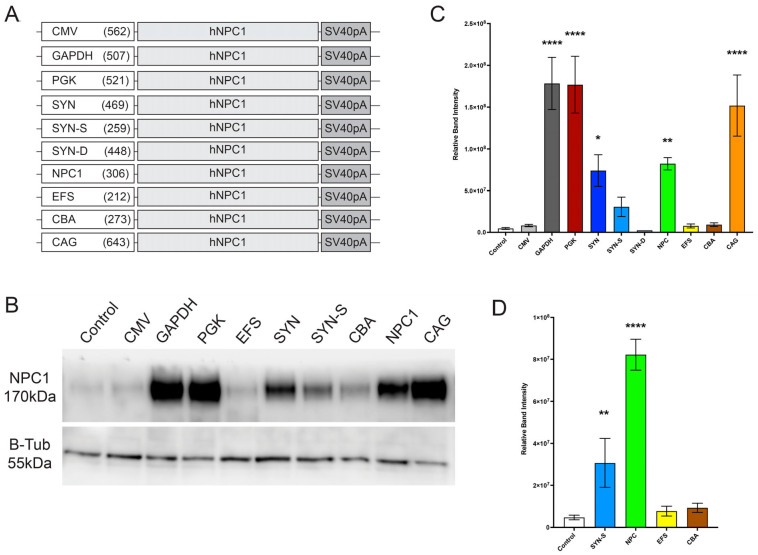
In vitro promoter comparison with human *NPC1* cDNA. (**A**) Representative figures of different promoters tested with size in base pairs (bp). (**B**) Western blot staining for NPC1 72 h post-transfection, demonstrating NPC1 levels in cell lysates. Staining of B-Tubulin used as loading controls. (**C**) Quantification of the relative NPC1 band, normalised to B-Tubulin intensity from transduced cells (*n* = 3 wells) for all promoters and (**D**) for promoters under 400 bp. One-way ANOVA with post-hoc Bonferroni correction, *n* = 3, error bars indicate ± SD. All significance shown is to control group. * p < 0.05, ** *p* < 0.01, **** *p* < 0.0001. A full list of *p*-values is reported in Supplementary File S1.

The same range of promoters were used to produce AAV expression plasmids carrying the human *NPC1* cDNA sequence (Figure 3A). To confirm functional expression of human *NPC1* (h*NPC1*), all expression plasmids were transfected into HEK293 cells and after 72 h a western blot of the cell lysate was performed using antibodies against NPC1 with beta-tubulin as the loading control (*n* = 3). Plasmids with GAPDH, PGK, SYN, NPC, and CAG produced a significant increase in NPC1 compared to endogenous levels in untransduced controls, and of the expected size of 170 kDa (Figure 3B) (all *p*-values are shown in Supplementary File S1). Of the promoters that were small enough for the genome size to remain below 4.7 Kb, the *NPC1* promoter provided the highest levels of expression (Figure 3C).

Next, we wanted to confirm the expression of these constructs in primary culture cells, as before for the reporter gene eGFP (Supplementary Figure S1), but this time driving *NPC1* gene expression. Therefore, viral preparations of AAV9-NPC1-*NPC1*, AAV9-SYN-h*NPC1*, and AAV9-CAG-h*NPC1* were produced. The vectors were applied to the primary cortical cell cultures prepared from the brains of E18 wild-type mice embryos and transduced with the three viral vectors at a MOI of 100,000 vg/cell. Immunofluorescence studies using the antibodies and stains described in Supplementary Figure S1 were performed, except for the GFP antibodies that were replaced with anti-NPC1 antibodies. Imaging by confocal microscopy confirmed that the NPC promoter drove NPC1 expression in both NeuN-labelled neurons and GFAP-labelled astrocytes (Supplementary Figure S2). The same was observed in cells transduced with AAV9-CAG-h*NPC1*, while cells transduced with AAV9-SYN-h*NPC1* only showed expression of NPC1 in NeuN-labelled neurons and not in GFAP-labelled astrocytes (Supplementary Figure S2).

### 3.4. The NPC Promoter Provides Enhanced NPC1 Expression, Attenuated Behavioural Deficits, and Improves Survival in the Npc1^nih^ Mouse Model following AAV9-Mediated Gene Therapy

**Figure 4:**
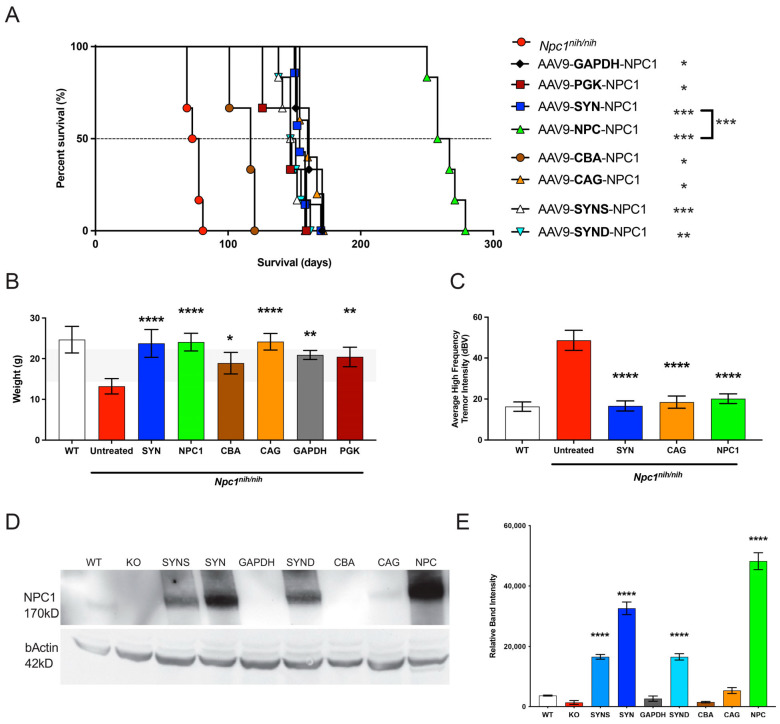
Evaluation of AAV9-h*NPC1* vectors with different promoters in *Npc1^nih^* mice. (**A**) Enhanced survival of *Npc1^nih/nih^* mice following AAV9-h*NPC1* treatment, with CBA promoter only showing partial rescue, SYN promoter showing doubling of lifespan, and surprisingly, the truncated NPC promoter extending the lifespan beyond all other tested promoters. All comparisons by Log-rank (Mantel–Cox) test, *n* = 6 either compared to untreated controls or between two selected groups. Dashed line is a reference line for 50% survival (**B**) normalisation of week 10 weights of *Npc1^nih/nih^* mice to wild-type levels achieved with various promoters. One-way ANOVA with post hoc Bonferroni correction, *n* = 6, error bars indicate ± SD. All significance shown is to control group. (**C**) Quantification of high frequency tremor analysis of *Npc1^nih/nih^* mice at 10 weeks treated P0 ICV with AAV9-h*NPC1* vector containing different promoters. One-way ANOVA with post hoc Bonferroni correction, *n* = 6, error bars indicate ± SD. (**D**) Western blot against NPC1 protein from half brain lysates of *Npc1^nih/nih^* mice, normalised to B-Actin treated with AAV9-h*NPC1* vectors containing different promoters. (**E**) Quantification of the relative NPC1 band, normalised to B-Actin intensity from half brain lysates (*n* = 3) for all promoters. One-way ANOVA with post hoc Bonferroni correction, *n* = 3, error bars indicate ± SD. All significance shown is to the untreated *Npc1^nih/nih^* control group. * *p* < 0.05, ** *p* < 0.01, *** *p* < 0.001, and **** *p* < 0.0001. A full list of *p*-values is reported in Supplementary File S1.

The following vectors and promoters were assessed for therapeutic efficacy in the *Npc1^nih^* mouse model: AAV9-GAPDH-h*NPC1*, AAV9-PGK-h*NPC1*, AAV9-NPC1-h*NPC1*, AAV9-CBA-h*NPC1*, AAV9-SYN-h*NPC1*, AAV9-SYN-S-h*NPC1*, AAV9-SYN-D-h*NPC1* AAV9-CBA-h*NPC1*, and AAV9-CAG-h*NPC1*. The vectors were administered at a dose of 1.5 × 10^11^ vector genomes via intracerebroventricular injection in P0–P1 newborn *Npc1^nih/nih^* mice (*n* = 6), as before [24]. Wild-type (*Npc1*^+/+^) mice and untreated *Npc1^nih/nih^* mice acted as age-matched controls. The experimental cohorts were assessed for survival, weight, tremor, and locomotor function by semi-quantitative gait analysis. A study of survival in treated mice showed that untreated *Npc1^nih/nih^* mice reached their humane endpoint at a median 75.5 days of age (Figure 4A). All the gene therapy-treated *Npc1^nih/nih^* mice experimental cohorts showed a significant increase in life span compared to the untreated mice (all *p*-values are shown in Supplementary File S1); AAV9-GAPDH-h*NPC1* (median survival 161 days), AAV9-PGK-h*NPC1* (median survival 147 days), AAV9-NPC1-h*NPC1* (median survival 262.5 days), AAV9-CBA-h*NPC1*(median survival 117 days), AAV9-SYN-h*NPC1* (median survival 154 days), AAV9-SYN-S-h*NPC1* (median survival 147.5 days), AAV9-SYN-D-h*NPC1* (median survival 149 days), AAV9-CBA-h*NPC1* (median survival 117 days), and AAV9-CAG-h*NPC1*(median survival 160 days). Therefore, the largest increase in survival was for AAV9-NPC1-h*NPC1* (Figure 4A).

At 10 weeks of age the untreated *Npc1^nih/nih^* mice had reached their humane endpoint and lost a significant amount of weight compared to the age-matched control *Npc1*^+/+^ mice (Figure 4B). However, all experimental cohorts that were treated with gene therapy vectors showed no significant reduction in body weights compared to the *Npc1^+/+^* mice at 10 weeks of age.

Assessment of tremor using an automated tremor sensor [24] revealed that the untreated *Npc1*^nih/nih^ mice showed the characteristic tremor compared to the wild-type *Npc1*^+/+^ mice (Figure 4C). However, this was normalised across the tested treatment groups—AAV9-SYN-h*NPC1*, AAV9-CAG-h*NPC1*, and AAV9-NPC1-h*NPC1*.

To determine how these data correlated with NPC1 expression, western blotting was performed on whole brain homogenates from *n* = 3 at 10 weeks (Figure 4D). Analysis of these western blots revealed a significant increase in NPC1 expression for AAV9-SYN-h*NPC1*, AAV9-SYN-D-h*NPC1*, AAV9-SYN-S-h*NPC1*, and AAV9-NPC1-h*NPC1* (Figure 4E). While high levels of CNS expression are to be expected with the SYN1 promoter, it was surprising that there was a higher level of NPC1 expression with the small truncated *NPC1* promoter (Figure 4E).

**Figure 5:**
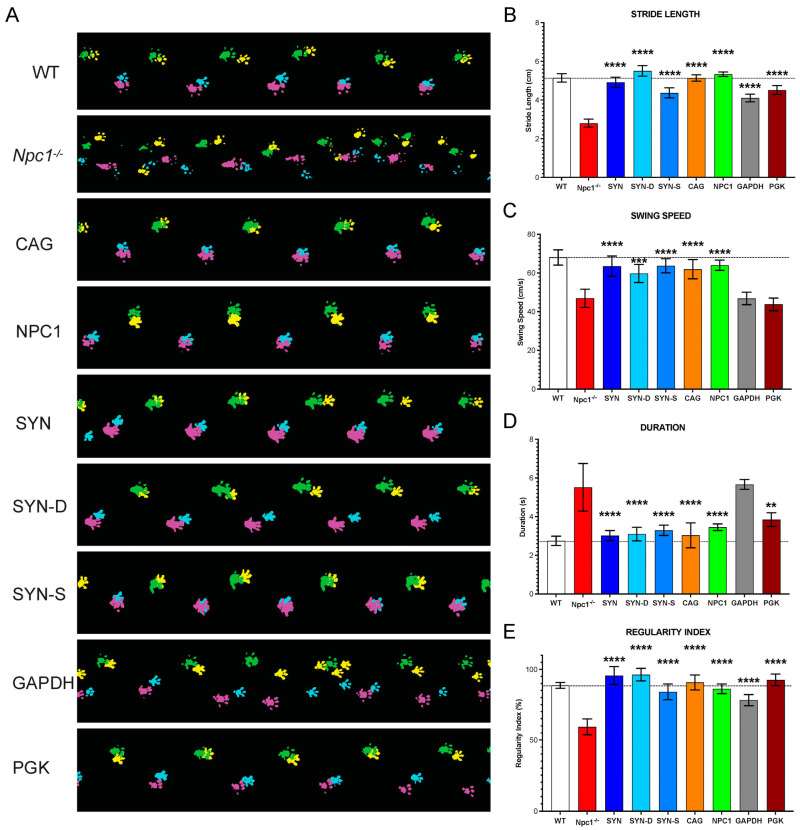
Gait analysis of *Npc1^nih^* mice treated with AAV9–h*NPC1* vectors with different promoters. (**A**) Graphical representation of paw prints (Yellow: Left-Front, Cyan: Right-Front, Pink: Right-Hind and Green: Left-Hind paws) captured during an average run on the *CatWalk* XT semi-automated gait analysis by mice at 10 weeks of age treated P0 ICV with AAV9-h*NPC1* vector containing different promoters. Quantification of four critical gait parameters affected in *Npc1^nih/nih^* mice: (**B**) Stride length: distance (cm) between successive paw placements, (**C**) Swing speed (cm/sec): speed of movement of paws between successive placements, (**D**) Duration: average time (sec) taken to complete a run across the recorded 20cm walkway and (**E**) Regularity index: Percentage (%) of normal step sequence patterns during uninterrupted run, measuring the degree of interlimb coordination. One-way ANOVA with post hoc Bonferroni correction, *n* = 6, error bars indicate ± SD. All significance shown is to the untreated *Npc1^nih/nih^* control group. ** *p* < 0.01, *** *p* < 0.001, and **** *p* < 0.0001. Dashed lines are a reference for mean WT value. A full list of *p*-values is reported in Supplementary File S1.

At 10 weeks of age all experimental groups were assessed for locomotor function using the CatwalkXT gait analysis system. An examination of the gait traces revealed a significant deterioration in the *Npc1*^nih/nih^ mice compared to the age-matched *Npc1*^+/+^ (Figure 5A). All treatment groups showed an improvement in gait, with the exception of the *Npc1^nih/nih^* mice treated with AAV9-GAPDH-h*NPC1*. Quantification of stride length and regularity index showed a significant improvement across all treatment groups, as compared to untreated controls, whereas AAV9-GAPDH-h*NPC1* and AAV9-PGK-h*NPC1*-treated animals did not show significant improvement in swing speed and AAV9-GAPDH-h*NPC1*-treated animals showed no significant improvements in duration of run. Together, our data show that most of the tested vectors attenuate locomotor deficits in the treated mice, with the exception of the AAV9-GAPDH-h*NPC1* and AAV9-PGK-h*NPC1*-treated mice, which show a lesser therapeutic effect.

### 3.5. The NPC Promoter Provides Differing Levels of Gene Expression in WT and NPC1-Deficient Mice

**Figure 6:**
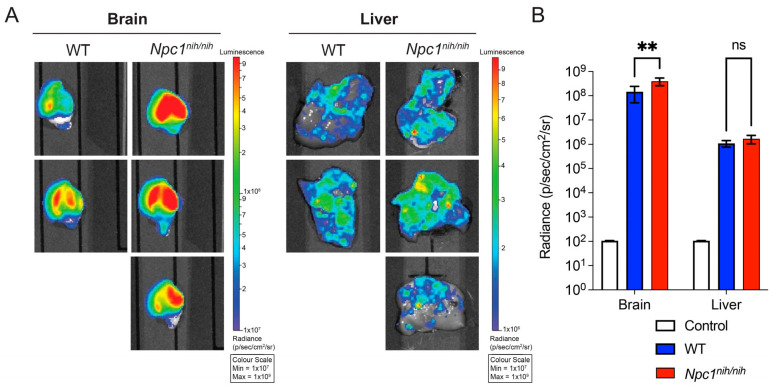
In vivo comparison of *NPC1* promoter activity in vivo in wild-type and *Npc1^nih/nih^* mice. (**A**) Comparison of luciferase expression in brains and livers of 10-week-old mice wild-type and *Npc1^nih/nih^* mice administered ICV neonatally with AAV9.NLSeGFP.2AFLuc under control of the NPC promoter. (**B**) Quantification of luciferase expression levels from analysed tissue in photons/second/cm^2^/steradian. Two-way ANOVA with post hoc Bonferroni correction, *n* = 3, error bars indicate ± SD. A full list of *p*-values is reported in Supplementary File S1. ** *p* < 0.01, ns (no significant difference).

Given that the NPC promoter did not show the highest levels of reporter gene expression in the wild-type mice (Figure 2) but showed significantly higher levels of expression when injected into the *Npc1^nih/nih^* mice, we further assessed whether a *Npc1* deficiency had any effect on the levels of transgene expression. We injected P0–1 wild-type and *Npc1*^nih^ mice (*n* = 3), with AAV9-NPC1-NLSeGFP.2A.FLuc at a dose of 1.5 × 10^11^ vector genomes via intracerebroventricular injection. The mice were then culled at 70 days and their brains and livers were analysed using the in vivo imaging system (IVIS) [29]. Analysis of radiance revealed a significantly higher radiance in the *Npc1*^nih^ as compared to the wild-type controls, indicating an overall higher expression of the reporter gene in the knock-out animals as compared to the wild-type animals (Figure 6)

### 3.6. The NPC Promoter Provides Enhanced NPC1 Expression and Significantly Attenuates Neuropathology in the Npc1^nmf164^ Mouse Model

Following the promising data obtained from successful survival and behavioural studies in the acute *Npc1*^nih^ mouse model, we next decided to compare the truncated *NPC1* promoter to a previously studied SYN1 promoter [16] in the slower progressing *Npc1^nmf164^* model and analysed the effect on attenuating neuropathology at the natural end-stage of the untreated *Npc1^nmf164^* mouse, 14 weeks [9].

For this, the *Npc1^nmf164^* mice were injected with AAV9-SYN-h*NPC1* and AAV9-NPC1-h*NPC1* vectors administered at a dose of 1.5 × 10^11^ vector genomes via intracerebroventricular injection in the P0–P1 newborn *Npc1^nih/nih^* mice (*n* = 3), as before [16] and compared with the untreated mice.

Immunohistochemical (IHC) analysis was performed in the primary somatosensory barrel field (S1BF), ventral-posterior medial and lateral (VPM/VPL) nuclei of the thalamus, and lobes VI and VII of the cerebellum (Cerebellum).

**Figure 7:**
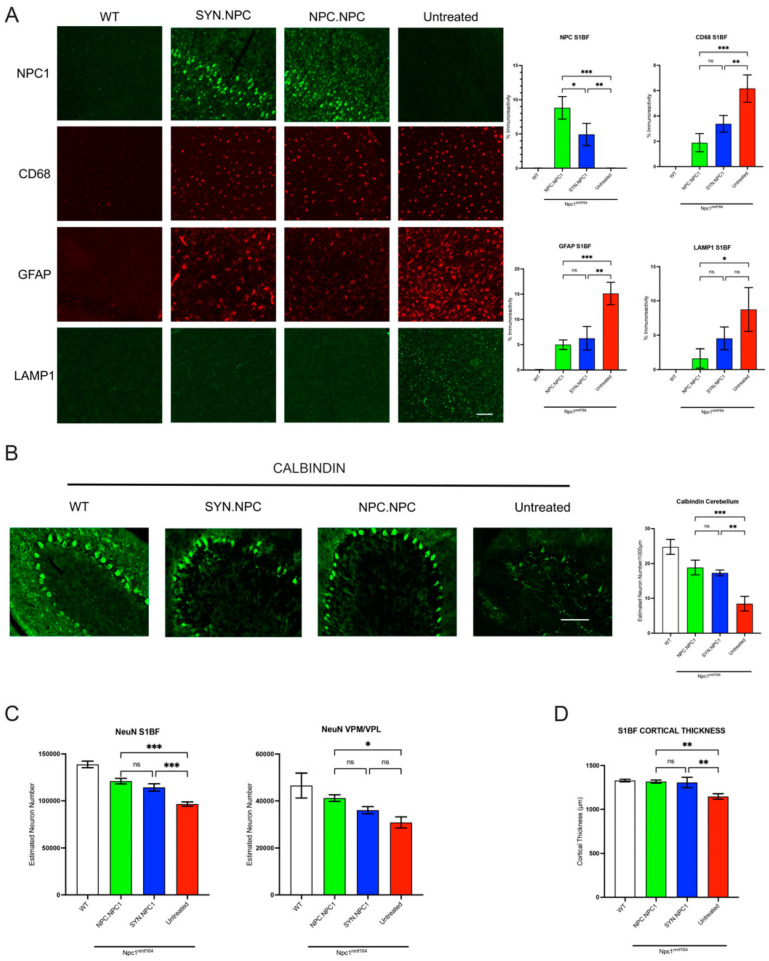
Efficacy of NPC1 and SYN promoters in attenuating neuropathology in *Npc1^nmf164^* mice. (**A**) Representative images and thresholding image analysis of immunofluorescence staining in the S1BF showing increase in NPC1 immunoreactivity and attenuation of microglial activation (CD68), astrocytosis (GFAP) and lysosomal burden (LAMP1) in AAV9-hNPC-treated brains as compared to untreated controls. Scale bar = 150 µm. (**B**) Representative images and manual stereological counts of Calbindin-positive Purkinje neurons in lobes VI–VII of the cerebellum showing significant neuroprotection in AAV9-hNPC1-treated brains as compared to untreated controls. Scale bar = 100 µm. (**C**) Stereological estimates of neuronal number in the S1BF and VPM/VPL in NeuN-stained brain sections showing significant neuroprotection in AAV9-h*NPC1*-treated brains as compared to untreated controls in the S1BF but only for AAV9-NPC-h*NPC1* in the VPM/VPL. (**D**) Cortical thickness measures in the S1BF showing significant attenuation of cortical atrophy in AAV9-h*NPC1*-treated brains as compared to untreated controls. One-way ANOVA with post hoc Bonferroni correction, *n* = 3, error bars indicate ± SD. All significance shown is to the untreated *Npc1^nih/nih^* control group. * *p* < 0.05, ** *p* < 0.01, *** *p* < 0.001 and where there is no significant difference (ns). A full list of *p*-values is reported in Supplementary File S1.

IHC staining for the hNPC1 protein showed very low levels in the WT and untreated *Npc1^nmf164^* mice but significantly increased levels in both the AAV9-SYN-h*NPC1* and AAV9-NPC1-h*NPC1*-treated *Npc1^nmf164^* mice (Figure 7A and Figure S3A,B). Further, the AAV9-NPC1-h*NPC1*-treated mice showed significantly higher levels of NPC1 expression than their AAV9-SYN-h*NPC1*-treated counterparts across all three regions (Figure 7A and Figure S3A,B).

As there is significant microglial and astrocyte activation across the brain at the end-stage in the untreated *Npc1^nmf164^* mice [9], we next analysed these phenotype stainings for microglia (CD68) and astrocytes (GFAP), as before. Our analysis revealed a significant increase in CD68 and GFAP staining across all regions analysed in the untreated *Npc1^nmf164^* mice, with significant attenuation of CD68 and GFAP immunoreactivity across the S1BF, VPM/VPL, and cerebellum in both the AAV9-SYN-h*NPC1* and AAV9-NPC1-h*NPC1*-treated *Npc1^nmf164^* mice, as compared to the untreated controls; however, these levels were not normalised to wild-type levels. Further, despite no statistical significance for CD68 or GFAP staining between the AAV9-SYN-h*NPC1* and AAV9-NPC1-h*NPC1*-treated *Npc1^nmf164^* mice, there was a consistent trend towards lower values in the AAV9-NPC1-h*NPC1*-treated animals (Figure 7A and Figure S3).

Next, we analysed brain sections stained for the lysosomal-associated membrane protein 1 (LAMP1), to assess lysosomal burden [16]. We found a significant increase in LAMP1 staining across S1BF, VPM/VPL, and the cerebellum in the untreated *Npc1^nmf164^* mice with significant attenuation of immunoreactivity in both the AAV9-SYN-h*NPC1* and AAV9-NPC1-h*NPC1*-treated *Npc1^nmf164^* mice across all regions (Figure 7A and Figure S3A,B).

Neuron loss is a significant feature of NP-C disease progression and has been documented in *Npc1^nmf164^* mice [9]. We therefore first stained for Purkinje cells via Calbindin staining [16] and showed a significant loss of Purkinje neurons in lobes VI–VII of the cerebellum, and there was a statistically significant rescue of this phenotype in the AAV9-SYN-h*NPC1* and AAV9-NPC1-h*NPC1*-treated mice (Figure 7B). We also assessed overall neuron loss in the S1BF and VPM/VPL by staining for the ubiquitous neuronal marker, NeuN, and performing unbiased stereological counting [35]. Our results showed a significant increase in NeuN-positive neurons in the S1BF of the AAV9-SYN-h*NPC1* and AAV9-NPC1-h*NPC1*-treated mice as compared to their untreated counterparts, but only the AAV9-NPC1-h*NPC1*-treated mice showed a significant increase in NeuN counts compared to the untreated mice in the VPM/VPL (Figure 7C). Lastly, both the AAV9-SYN-h*NPC1* and AAV9-NPC1-h*NPC1*-treated mice showed a significant rescue in cortical atrophy as compared to the untreated controls (Figure 7D). 

## 4. Discussion

NP-C is a prematurely fatal neurodegenerative lysosomal storage disorder. Despite a plethora of preclinical therapies being tested for NP-C, only one drug, miglustat, has been approved for clinical therapy. However, miglustat slows disease progression and extends life span, but it does not halt disease progression [12,13]. Therefore, there is a significant need for readily translatable preclinical therapies for NP-C. With the recent regulatory approval of Zolgensma for spinal muscular atrophy [14,15], there is greater hope for a similar approach to be successful for other neurological disorders, including NP-C. While gene therapy has previously been tested in preclinical models of NP-C [16,17,18,19], these approaches are yet to be successfully translated into the clinic. A major hurdle in designing an AAV vector for NP-C is the size of the human *NPC1* gene. The large size of the gene means less space for other crucial elements such as the promoter, polyA signal, or regulatory sequences. Exceeding the limited packaging capacity of AAV vectors (4.7 Kb) could lead to truncation and packaging defects during vector manufacturing [21].

While techniques using spilt vectors, overlapping dual vectors [36], or development of minigenes [37] exist to reduce the size of the transgene, another avenue to be pursued would be the choice of promoter. The promoter sequence not only determines the level of transgene expression as well as tissue specificity but may also significantly influence the overall size of the gene product to be packaged [24,38,39,40].

Therefore, given the need for a small, ubiquitous, and efficient promoter for *NPC1*, we compared various promoters, including CMV (cytomegalovirus) promoter/enhancer, EFS (elongation factor 1a), GAPDH (glyceraldehyde-3-phosphate dehydrogenase), mPGK (phosphoglycerate kinase), CBA (chicken β-actin), CAG (chicken β-actin promoter with CMV enhancer), Synapsin-1, and a truncated *NPC1* promoter. Of these, the EFS, CBA, and *NPC1* promoters were the only promoters under the desired 400 bp size. Further, we also tested a neuron de-targeted Synapsin-1 (SYN-D) promoter and a shortened Synapsin-1 (SYN-S) promoter under 400 bp. While the focus was to find an efficient promoter under 400 bp, it was also crucial to compare these with more commonly used, but larger promoters, to see if this efficacy could be matched.

Our data revealed that when tested in vitro, the small truncated *NPC1* promoter showed the highest level of expression of both a reported eGFP as well as the h*NPC1* gene, among the promoters under 400 bp. While this expression was lower than other larger promoters including CAG, PGK, and GAPDH, these were still unexpectedly significant results for this novel promoter (Figure 1 and Figure 3). Furthermore, unlike Synapsin-1, which is a predominantly neuronal promoter, the *NPC1* promoter was also expressed in astrocytes in culture, similar to the CAG promoter, indicating its ubiquitous expression in the CNS (Supplementary Figures S1 and S2). In vivo injections of viral preparations into the ventricles of the neonatal wild-type mice also showed high levels of brain expression, even when compared to a strong neuronal promoter—synapsin-1 (Figure 2).

To test therapeutic efficacy, we then injected the various viral preparations into the neonatal *Npc1^nih^* mice, and here, the *NPC1* promoter showed a comparable effect in attenuating weight loss and the tremor phenotype at 10 weeks, as compared to most promoters. However, in terms of overall survival and NPC1 protein expression, there were significantly improved outcomes with the *NPC1* promoter, as compared to any other promoter tested (Figure 4 and Figure 5). The significant result of this novel, small promoter is perhaps explained by the differential expression observed when AAV viral preparations were injected into the wild-type versus the *Npc1*-deficient mice (Figure 7), indicating that the *NPC1* promoter drives greater gene expression in the deficient model. However, the mechanism underlying such differential expression awaits further investigation. Moreover, the possibility of additional gene interactions arising from the expression of NPC1 from this truncated endogenous promoter and their downstream epigenetic and translational consequences must also be elucidated.

Lastly, we investigated whether the *NPC1* promoter is effective in reducing neuropathological phenotypes in a more slowly progressing mouse model, the *Npc1^nmf164^* mouse [9], comparing it to the previously described Synapsin-1 (SYN) promoter [16]. In contrast to the NPC1 null *Npc1^nih^* model, the *Npc1^nmf164^* mice express low levels of the misfolded NPC1 protein, similar to the majority of NPC patients. Our data show a significantly higher level of NPC1 protein expression driven by the *NPC1* promoter compared to the SYN promoter, consistent with the previous western blot analysis (Figure 4). Further, there was an overall comparable attenuation of microglial activation, astrocytosis, and lysosomal burden between NPC1 and SYN promoters. However, while the SYN.hNPC1-treated mice did not show a significant rescue of neurons in the deeper-lying thalamus, the NPC.h*NPC1*-treated mice showed a significant rescue in this region, possibly due to the overall higher level of NPC1 expression in the brain. These findings, along with significant neuron rescue in the S1BF and Purkinje cells in the cerebellum, show that the novel *NPC1* promoter is overall more effective in preventing the onset of neuropathological features, as compared to the SYN promoter.

Taken together, our data for the first time describe a novel small, truncated endogenous *NPC1* promoter that is efficient in driving gene expression both in vitro and in vivo, with significantly improved survival and NPC1 expression in the knockout *Npc1^nih^* mouse and overall effective reduction in neuropathology in the *Npc1^nmf164^* mouse models of NP-C that carry a missense point mutation. Unexpectedly, this promoter drives gene expression at a higher level in the NPC1-deficient mice, implying a potential feedback loop, whose mechanism awaits further validation.

Taken together, our studies corroborate our previous data, demonstrating that AAV-mediated gene therapy administered ICV has significant therapeutic potential for NP-C, and that an optimised AAV design strategy must also take into account the DNA regulatory elements as well as the overall packaging restrictions depending on the gene of interest. The translation of pre-clinical gene therapy studies to successful clinical trials for other neurodegenerative conditions means that this is an opportune moment to further pursue the development of gene therapy for NP-C.

## Data Availability

All data are available on request.

## Supplementary Materials

The following supporting information can be downloaded at: https://www.mdpi.com/article/10.3390/cells12121619/s1. Figure S1. Verification of neuronal and glial cell expression of NLSeGFP reporter gene in primary brain cultures in vitro. (A) Co-staining of primary brain cultures transduced with AAV9 vectors expressing nuclear localised eGFP reporter gene driven by the SYN, CAG or *NPC1* promoter. Neuronal marker (NeuN, Red) and astrocyte marker (GFAP, Red) indicate cell type and eGFP (Green) demonstrates reporter gene expression with nuclear marker DAPI (Blue). White arrows indicate neuronal and glial cells that positively express the eGFP reporter gene. Scale bar = 50 µm. Figure S2. Verification of neuronal and glial cell expression of *NPC1* reporter gene in primary brain cultures in vitro. (A) Co-staining of primary brain cultures transduced with AAV9 vectors expressing h*NPC1* gene driven by the SYN, CAG or *NPC1* promoter. Neuronal marker (NeuN, Red) and astrocyte marker (GFAP, Red) indicate cell type and NPC1 (Green) demonstrates NPC1 expression with nuclear marker DAPI (Blue). White arrows indicate neuronal and glial cells that positively express the eGFP reporter gene. Scale bar = 50 µm. Figure S3. Efficacy of NPC1 and SYN promoters in attenuating thalamic and cerebellar neuropathology in *Npc1^nih^* mice (A) Representative images and thresholding image analysis of immunofluorescence staining in the VPM/VPL showing increase in NPC1 immunoreactivity and attenuation of microglial activation (CD68), astrocytosis (GFAP) and lysosomal burden (LAMP1) in AAV9-h*NPC1* treated brains as compared to untreated controls.(B) Representative images of lobes VII of Cerebellum and thresholding image analysis of immunofluorescence staining in lobes VI-VII of Cerebellum showing increase in NPC1 immunoreactivity and attenuation of microglial activation (CD68), astrocytosis (GFAP) and lysosomal burden (LAMP1) in AAV9-h*NPC1* treated brains as compared to untreated controls. Scale bar = 150 µm. One-way ANOVA with post-hoc Bonferroni correction, *n* = 3, error bars indicate ± SD. All significance shown is to untreated *Npc1^nih/nih^* control group. * *p* < 0.05, ** *p* < 0.01, *** *p* < 0.001, **** *p* < 0.0001. Full list of *p*-values are reported in Supplementary File S1. Supplementary File S1—*p*-values of all figures. Supplementary File S2—Promoter sequences used and plasmid map.
