# Supplementary material for: A Novel Small NPC1 Promoter Enhances AAV-Mediated Gene Therapy in Mouse Models of Niemann–Pick Type C1 Disease
Source: Cells. 2023 Jun 13;12(12):1619. doi: 10.3390/cells12121619 (PMC10296851; doi:10.3390/cells12121619)
Supplement: Supplementary file 1 [file cells-12-01619-s001.zip › cells-2323087-supplementary.pdf]

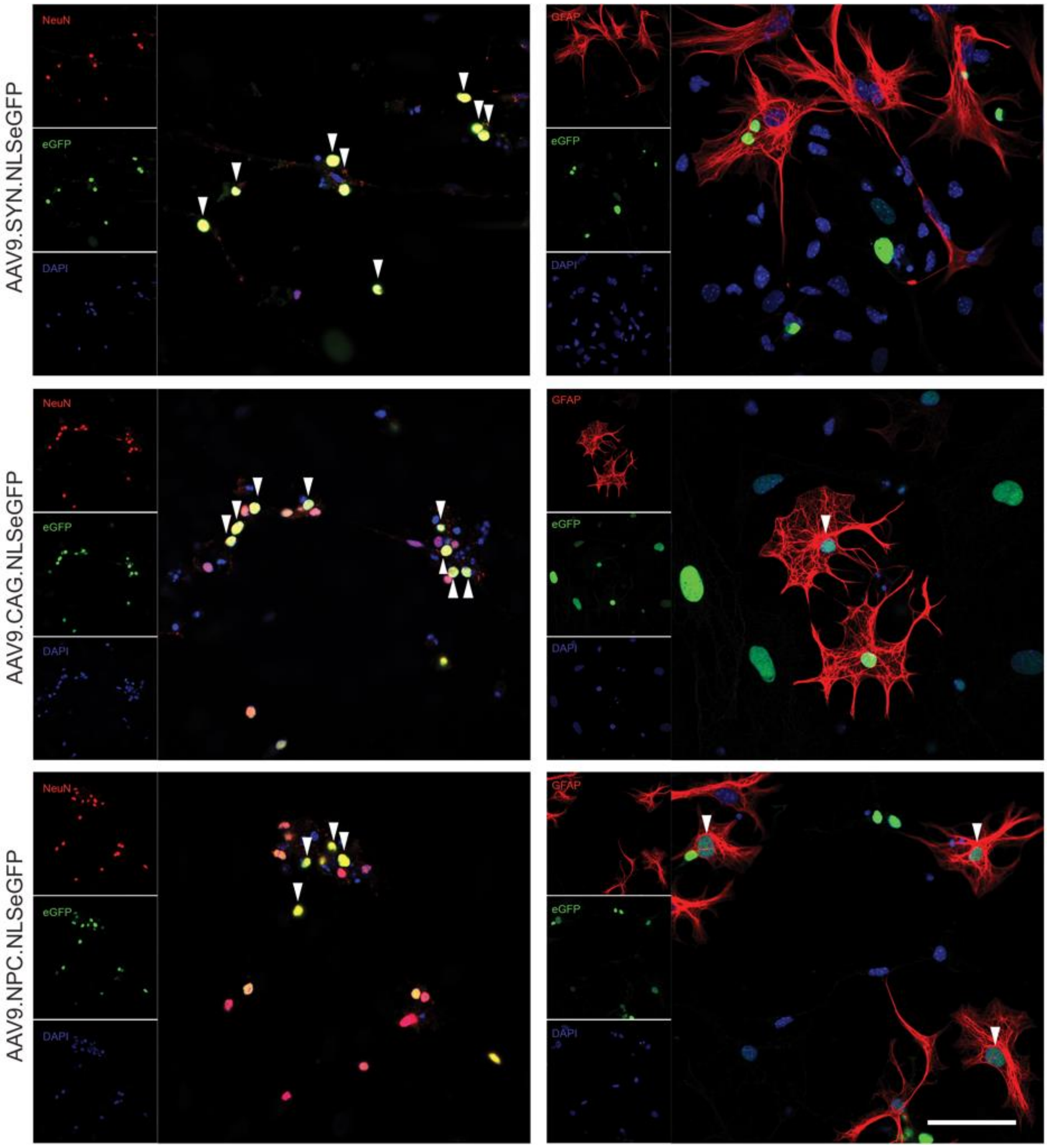

Figure S1: Verification of neuronal and glial cell expression of NLSeGFP reporter gene in primary brain cultures *in vitro*. (A) Co-staining of primary brain cultures transduced with AAV9 vectors expressing nuclear localised eGFP reporter gene driven by the SYN, CAG or NPC1 promoter. Neuronal marker (NeuN, Red) and astrocyte marker (GFAP, Red) indicate cell type and eGFP (Green) demonstrates reporter gene expression with nuclear marker DAPI (Blue). White arrows indicate neuronal and glial cells that positively express the eGFP reporter gene. Scale bar = 50µm

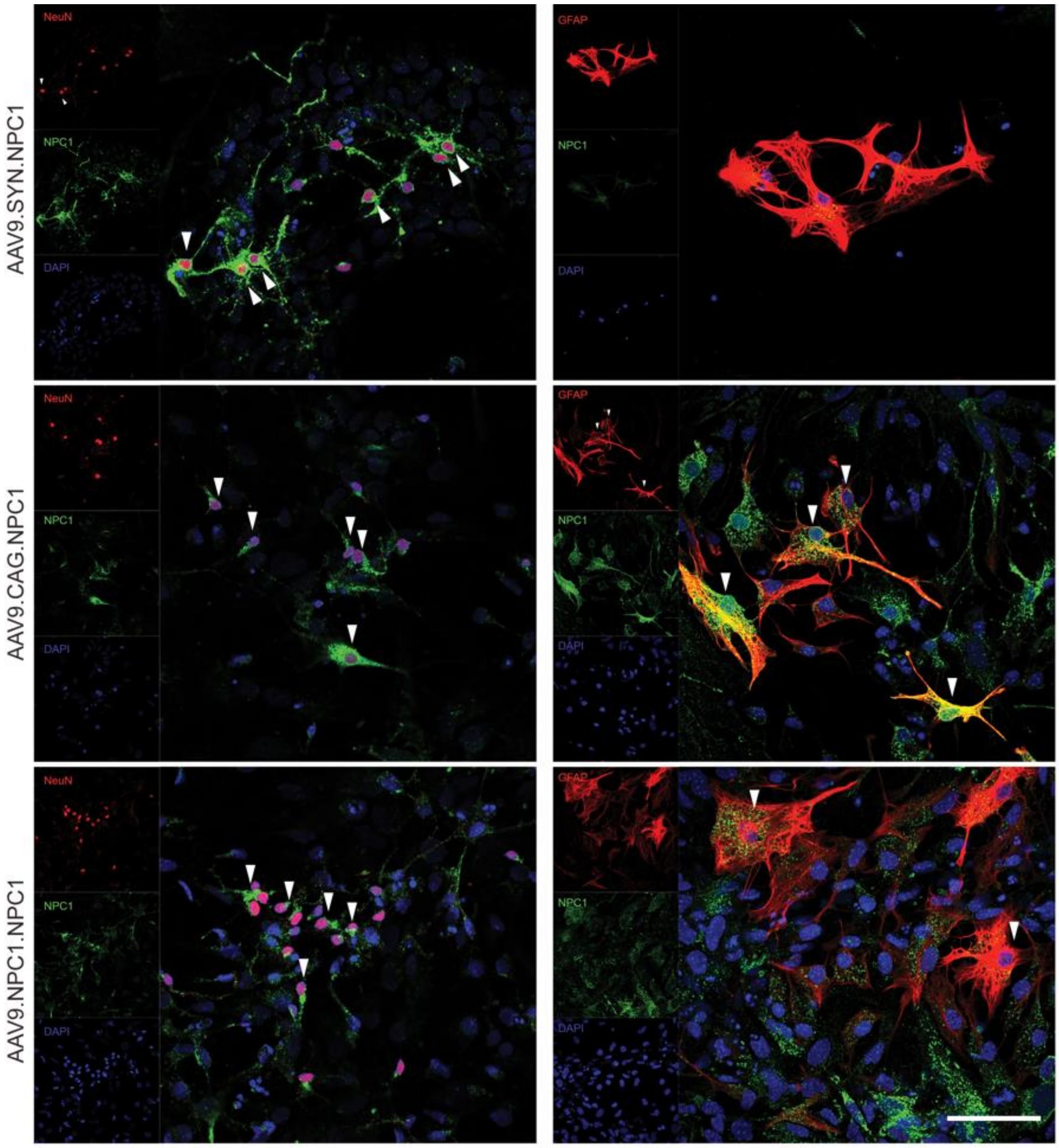

Figure S2: Verification of neuronal and glial cell expression of NPC1 reporter gene in primary brain cultures *in vitro*. (A) Co-staining of primary brain cultures transduced with AAV9 vectors expressing hNPC1 gene driven by the SYN, CAG or NPC1 promoter. Neuronal marker (NeuN, Red) and astrocyte marker (GFAP, Red) indicate cell type and NPC1 (Green) demonstrates NPC1 expression with nuclear marker DAPI (Blue). White arrows indicate neuronal and glial cells that positively express the eGFP reporter gene. Scale bar = 50μm

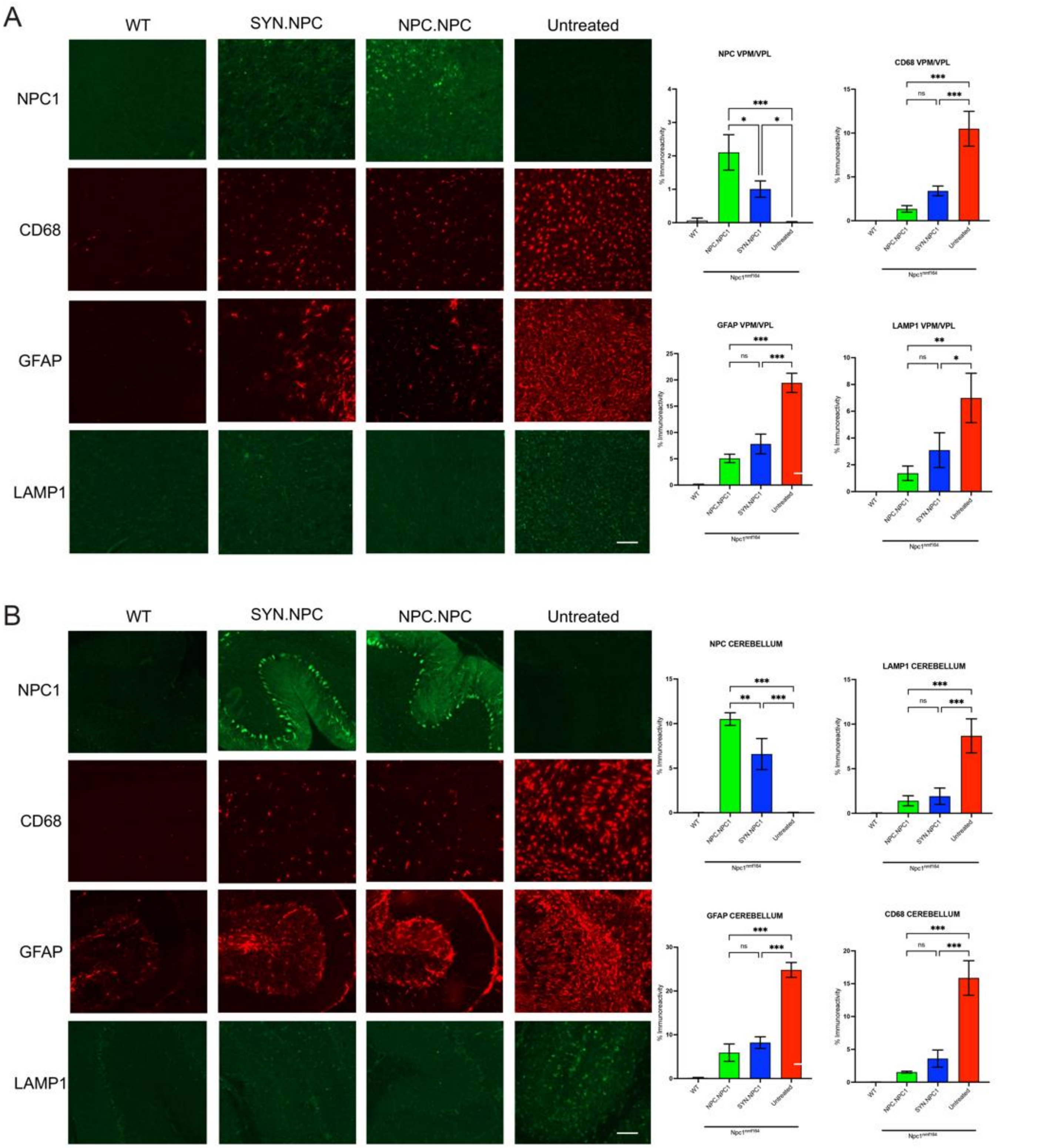

Figure S3: Efficacy of NPC1 and SYN promoters in attenuating thalamic and cerebellar neuropathology in *$Npc1^{nih}$* mice (A) Representative images and thresholding image analysis of immunofluorescence staining in the VPM/VPL showing increase of NPC1 immunoreactivity and attenuation of microglial activation (CD68), astrocytosis (GFAP) and lysosomal burden (LAMP1) in AAV9-hNPC1 treated brains as compared to untreated controls.(B) Representative images of lobes VII of Cerebellum and thresholding image analysis of immunofluorescence staining in lobes VI-VII of Cerebellum showing increase of NPC1 immunoreactivity and attenuation of microglial activation (CD68), astrocytosis (GFAP) and lysosomal burden (LAMP1) in AAV9-hNPC1 treated brains as compared to untreated controls. Scale bar = 150µm. One-way ANOVA with *post-hoc* Bonferroni

correction, n=3, error bars indicate ± SD. All significance shown is to untreated *Npc1*$^{nih/nih}$ control group. * $p < 0.05$, ** $p < 0.01$, *** $p < 0.001$, **** $p < 0.0001$. Full list of p-values are reported in Supplementary File 1.
