# Supplementary material for: A Novel Small NPC1 Promoter Enhances AAV-Mediated Gene Therapy in Mouse Models of Niemann–Pick Type C1 Disease
Source: Cells. 2023 Jun 13;12(12):1619. doi: 10.3390/cells12121619 (PMC10296851; doi:10.3390/cells12121619)

CAG PROMOTER
5’GCGTTACATAACTTACGGTAAATGGCCCGCCTGGCTGACCGCCCAACGACCCCCGCCCATTGACGT
CAATAATGACGTATGTTCCCATAGTAACGCCAATAGGGACTTTCCATTGACGTCAATGGGTGGAGTA
TTTACGGTAAACTGCCCACTTGGCAGTACATCAAGTGTATCATATGCCAAGTACGCCCCCTATTGACG
TCAATGACGGTAAATGGCCCGCCTGGCATTATGCCCAGTACATGACCTTATGGGACTTTCCTACTTG
GCAGTACATCTACGTATTAGTCATCGCTATTACCATGGTCGAGGTGAGCCCCACGTTCTGCTTCACTC
TCCCCATCTCCCCCCCCTCCCCACCCCCAATTTTGTATTTATTTATTTTTTAATTATTTTGTGCAGCGAT
GGGGGCGGGGGGGGGGGGGGGGCGCGCGCCAGGCGGGGCGGGGCGGGGCGAGGGGCGGGGC
GGGGCGAGGCGGAGAGGTGCGGCGGCAGCCAATCAGAGCGGCGCGCTCCGAAAGTTTCCTTTTAT
GGCGAGGCGGCGGCGGCGGCGGCCCTATAAAAAGCGAAGCGCGCGGCGGGCG**3’**

CBA PROMOTER
5’CCACGTTCTGCTTCACTCTCCCCATCTCCCCCCCCTCCCCACCCCCAATTTTGTATTTATTTATTTTTT
AATTATTTTGTGCAGCGATGGGGGCGGGGGGGGGGGGGGGGCGCGCGCCAGGCGGGGCGGGGCGG
GGCGAGGGGCGGGGCGGGGCGAGGCGGAGAGGTGCGGCGGCAGCCAATCAGAGCGGCGCGCTC
CGAAAGTTTCCTTTTATGGCGAGGCGGCGGCGGCGGCGGCCCTATAAAAAGCGAAGCGCGCGGCG
G**3’**

CMV PROMOTER
5’TTAATAGTAATCAATTACGGGGTCATTAGTTCATAGCCCATATATGGAGTTCCGCGTTACATAACTT
ACGGTAAATGGCCCGCCTGGCTGACCGCCCAACGACCCCCGCCCATTGACGTCAATAATGACGTATG
TTCCCATAGTAACGCCAATAGGGACTTTCCATTGACGTCAATGGGTGGAGTATTTACGGTAAACTGC
CCACTTGGCAGTACATCAAGTGTATCATATGCCAAGTACGCCCCCTATTGACGTCAATGACGGTAAA
TGGCCCGCCTGGCATTATGCCCAGTACATGACCTTATGGGACTTTCCTACTTGGCAGTACATCTACGT
ATTAGTCATCGCTATTACCATGGTGATGCGGTTTTGGCAGTACATCAATGGGCGTGGATAGCGGTTT
GACTCACGGGGATTTCCAAGTCTCCACCCCATTGACGTCAATGGGAGTTTGTTTTGGCACCAAAATC
AACGGGACTTTCCAAAATGTCGTAACAACTCCGCCCCATTGACGCAAATGGGCGGTAGGCGTGTAC
GGTGGGAGGTCTATATAAGCAGAGCT**3’**

EFS PROMOTER
5’GGGCAGAGCGCACATCGCCCACAGTCCCCGAGAAGTTGGGGGGAGGGGTCGGCAATTGAACCG
GTGCCTAGAGAAGGTGGCGCGGGGTAAACTGGGAAAGTGATGTCGTGTACTGGCTCCGCCTTTTTC
CCGAGGGTGGGGGAGAACCGTATATAAGTGCAGTAGTCGCCGTGAACGTTCTTTTTCGCAACGGGT
TTGCCGCCAGAACACAG**3’**

GAPDH PROMOTER
5’TCGAGGATATCAGTTCCCCAACTTTCCCGCCTCTCAGCCTTTGAAAGAAAGAAAGGGGAGGGGGC
AGGCCGCGTGCAGCCGCGAGCGGTGCTGGGCTCCGGCTCCAATTCCCCATCTCAGTCGTTCCCAAAG
TCCTCCTGTTTCATCCAAGCGTGTAAGGGTCCCCGTCCTTGACTCCCTAGTGTCCTGCTGCCCACAGT
CCAGTCCTGGGAACCAGCACCGATCACCTCCCATCGGGCCAATCTCAGTCCCTTCCCCCCTACGTCGG
GGCCCACACGCTCGGTGCGTGCCCAGTTGAACCAGGCGGCTGCGGAAAAAAAAAAGCGGGGAGAA
AGTAGGGCCCGGCTACTAGCGGTTTTACGGGCGCACGTAGCTCAGGCCTCAAGACCTTGGGCTGGG
ACTGGCTGAGCCTGGCGGGAGGCGGGGTCCGAGTCACCGCCTGCCGCCGCGCCCCCGGTTTCTATA
AATTGAGCCCGCAGCCTCCCGCTTCGCTCTCTGCTCCTCCTG**3’**

PGK PROMOTER

5’AATTCCCACGGGGTTGGGGTTGCGCCTTTTCCAAGGCAGCCCTGGGTTTGCGCAGGGACGCGGCT
GCTCTGGGCGTGGTTCCGGGAAACGCAGCGGCGCCGACCCTGGGTCTCGCACATTCTTCACGTCCG
TTCGCAGCGTCACCCGGATCTTCGCCGCTACCCTTGTGGGCCCCCCGGCGACGCTTCCTGCTCCGCCC
CTAAGTCGGGAAGGTTCCTTGCGGTTCGCGGCGTGCCGGACGTGACAAACGGAAGCCGCACGTCTC
ACTAGTACCCTCGCAGACGGACAGCGCCAGGGAGCAATGGCAGCGCGCCGACCGCGATGGGCTGT
GGCCAATAGCGGCTGCTCAGCGGGGCGCGCCGAGAGCAGCGGCCGGGAAGGGGCGGTGCGGGA
GGCGGGGTGTGGGGCGGTAGTGTGGGCCCTGTTCCTGCCCGCGCGGTGTTCCGCATTCTGCAAGCC
TCCGGAGCGCACGTCGGCAGTCGGCTCCCTCGTTGACCGAATCACCGACCTCTCTCCCCAGG**3’**

SYN (SYNAPSIN1) PROMOTER
5’CTGCAGAGGGCCCTGCGTATGAGTGCAAGTGGGTTTTAGGACCAGGATGAGGCGGGGTGGGGG
TGCCTACCTGACGACCGACCCCGACCCACTGGACAAGCACCCAACCCCCATTCCCCAAATTGCGCAT
CCCCTATCAGAGAGGGGGAGGGGAAACAGGATGCGGCGAGGCGCGTGCGCACTGCCAGCTTCAGC
ACCGCGGACAGTGCCTTCGCCCCCGCCTGGCGGCGCGCGCCACCGCCGCCTCAGCACTGAAGGCGC
GCTGACGTCACTCGCCGGTCCCCCGCAAACTCCCCTTCCCGGCCACCTTGGTCGCGTCCGCGCCGCC
GCCGGCCCAGCCGGACCGCACCACGCGAGGCGCGAGATAGGGGGGCACGGGCGCGACCATCTGC
GCTGCGGCGCCGGCGACTCAGCGCTGCCTCAGTCTGCGGTGGGCAGCGGAGGAGTCGTGTCGTGC
CTGAGAGCGCAG**3’**

SYN-S (Shortened Synapsin I promoter) PROMOTER
5’TTCGCCCCCGCCTGGCGGCGCGCGCCACCGCCGCCTCAGCACTGAAGGCGCGCTGACGTCACTCG
CCGGTCCCCCGCAAACTCCCCTTCCCGGCCACCTTGGTCGCGTCCGCGCCGCCGCCGGCCCAGCCGG
ACCGCACCACGCGAGGCGCGAGATAGGGGGGCACGGGCGCGACCATCTGCGCTGCGGCGCCGGC
GACTCAGCGCTGCCTCAGTCTGCGGTGGGCAGCGGAGGAGTCGTGTCGTGCCTGAGAGCGCAG**3’**

SYN-D (Neuron de-targeted Synapsin1) PROMOTER
5’CTGCAGAGGGCCCTGCGTATGAGTGCAAGTGGGTTTTAGGACCAGGATGAGGCGGGGTGGGGG
TGCCTACCTGACGACCGACCCCGACCCACTGGACAAGCACCCAACCCCCATTCCCCAAATTGCGCAT
CCCCTATCAGAGAGGGGGAGGGGAAACAGGATGCGGCGAGGCGCGTGCGCACTGCCAGCTTCGCC
CCCGCCTGGCGGCGCGCGCCACCGCCGCCTCAGCACTGAAGGCGCGCTGACGTCACTCGCCGGTCC
CCCGCAAACTCCCCTTCCCGGCCACCTTGGTCGCGTCCGCGCCGCCGCCGGCCCAGCCGGACCGCAC
CACGCGAGGCGCGAGATAGGGGGGCACGGGCGCGACCATCTGCGCTGCGGCGCCGGCGACTCAG
CGCTGCCTCAGTCTGCGGTGGGCAGCGGAGGAGTCGTGTCGTGCCTGAGAGCGCAG**3’**

NPC1 PROMOTER
5’CGAGCCAGACTCCATAAGTCCCGCGCCTGGCCCCCGGGGATTGCAGGGGCTGAGGAGAAGGGCA
ACACGGGGACCTTGAAGCGGGGTCGCGGCGGCGCCCCAGCCCGGGCCAGGGAGTCCCGGCAGCG
GCACCTCCCAGAAAGGGCGGAGCCGACGACGCCTTCTTCCTTCCTGACCGGCGCGCGCAGCCTGCT
GCCGCGGTCAGCGCCTGCTCCTGCTCCTCCGCTCCTCCTGCGCGGGGTGCTGAAACAGCCCGGGGA
AGTAGAGCCGCCTCCGGGGAGCCCAACCAGCCGAACGCCGCCGGCGT**3’**

pAAV9.NPC1.hNPC1.sv40pa

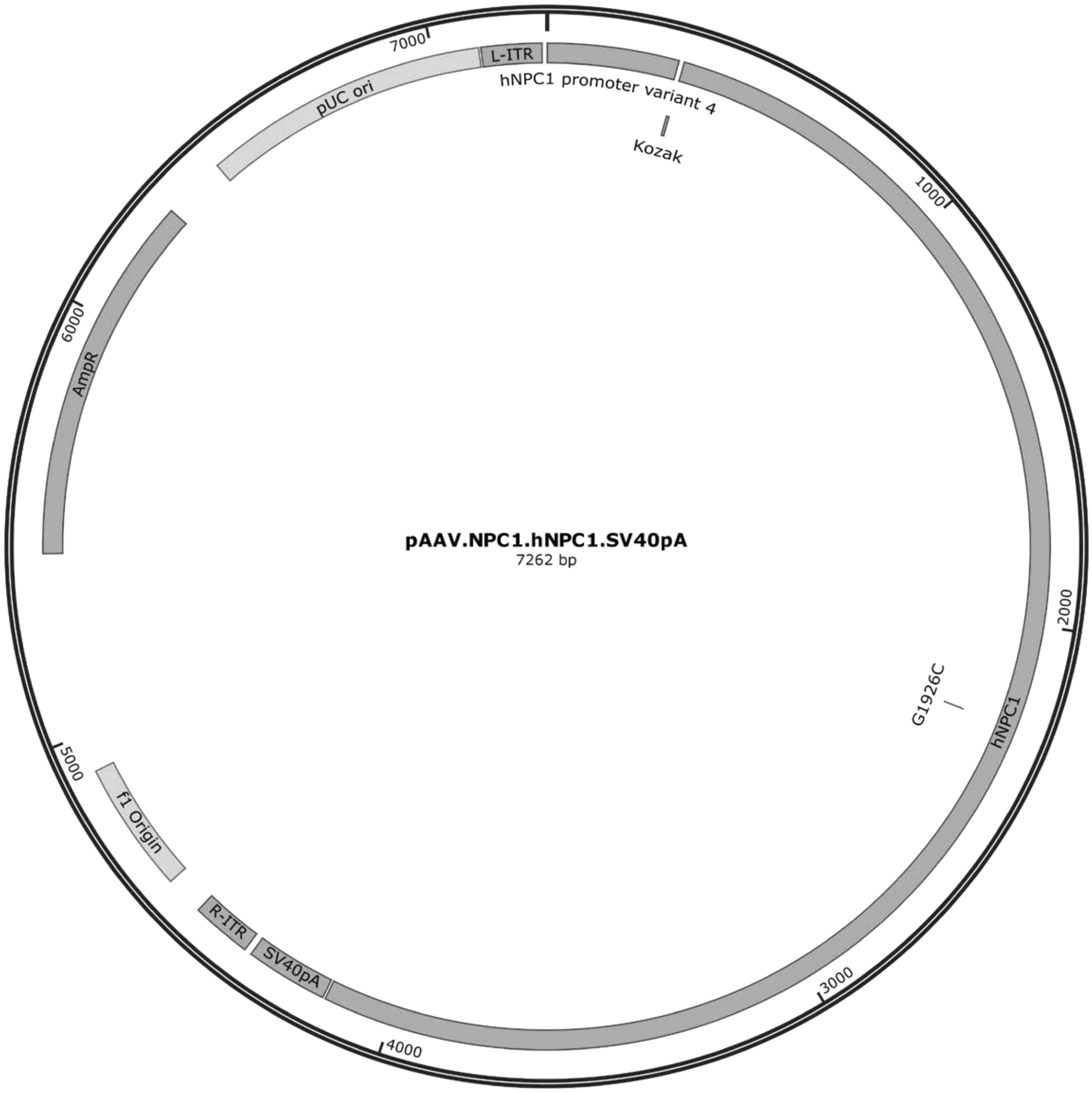

Supplement: Supplementary file 1 [file cells-12-01619-s001.zip › File S2 - PROMOTER SEQUENCES.pdf]
